# Development of an error‐detection examination for conservative dentistry education

**DOI:** 10.1002/cre2.250

**Published:** 2019-11-22

**Authors:** Masahiro Yoneda, Kazuhiko Yamada, Toshio Izumi, Etsuko Matsuzaki, Michito Maruta, Junko Hatakeyama, Hiromitsu Morita, Takashi Tsuzuki, Hisashi Anan, Takao Hirofuji

**Affiliations:** ^1^ Section of General Dentistry, Department of General Dentistry Fukuoka Dental College Fukuoka Japan; ^2^ Section of Operative Dentistry and Endodontology, Department of Odontology Fukuoka Dental College Fukuoka Japan; ^3^ Section of Bioengineering, Department of Dental Engineering Fukuoka Dental College Fukuoka Japan; ^4^ The Center for Visiting Dental Service, Department of General Dentistry Fukuoka Dental College Fukuoka Japan; ^5^ Section of Removable Prosthodontics, Department of Oral Rehabilitation Fukuoka Dental College Fukuoka Japan

**Keywords:** conservative dentistry, error‐detection examination, evaluation, questionnaire survey

## Abstract

**Objective:**

For dental students, textbooks and lectures provide basic knowledge, and simulated and actual clinical training provide learning in technical and communication skills. At our college, conservative dentistry is taught in the third and fourth years of a 6‐year undergraduate degree. Clinical training is undertaken subsequently in the fifth year and includes cavity preparation and composite resin filling tasks. However, despite the clinical importance of a full understanding surrounding these procedures, sixth‐year students occasionally provide incorrect answers regarding these procedures in assessments. Although they demonstrated a basic understanding of the procedures, they may have forgotten the acquired knowledge during their clinical training. Therefore, we developed an error‐detection examination to evaluate and improve fifth‐year students' knowledge.

**Methods:**

Written detailed treatment procedures for standardized, typical, cases were presented to students. Some critical steps were intentionally written incorrectly, and students had to identify and correct these. After correcting the steps, students gave a presentation to their peers on their corrections. This was followed by a summary of the correct answers and a short lecture by the teacher. Students then completed a questionnaire investigating their experience of the examination.

**Results:**

Students misunderstood some key treatment steps, such as pretreatment of composite resin filling, amalgam removal, and ceramic inlay fitting. The questionnaire revealed that this method of testing applied knowledge was new to students and helped them to identify knowledge gaps. The test also increased their motivation to study conservative dentistry. Students were open to taking similar tests in different areas.

**Conclusion:**

Although conservative dentistry is a basic field of dental treatment, mistakes in treatment can lead to early treatment failure or reduce the lifetime of a restored tooth. Therefore, students need to have a deep understanding of procedures. Error‐detection examinations may help students identify knowledge gaps and provide useful feedback to teachers to identify areas that they should stress in earlier years.

## 1 INTRODUCTION

Conservative dentistry is a basic field of clinical dentistry, and in this discipline, it is important to teach both the prevention of dental caries and how to maintain restorations over a long period (Staehle, Wolff, & Frese, 2015). A comprehensive understanding of conservative dentistry is therefore required for clinical training as an undergraduate and also to provide high‐quality care to patients after graduation. Within Japan, graduating dentists are required to demonstrate sufficient knowledge across dentistry by passing the Japan National Examination (JNE) before being eligible to become a practicing dentist (Komabayashi & Bird, 2005).

Emerging concepts in conservative dentistry are examined within the JNE as well as within undergraduate curricula, and students are therefore required to keep up to date with progress in this field.

For those studying dentistry, textbooks and lectures are traditionally the source of basic knowledge with technical and communication skills being gained from simulation laboratories and supervised clinical training. The undergraduate degree at Fukuoka Dental College is completed in 6 years, with preliminary conservative dentistry taught, and their knowledge assessed, in the third and fourth years. Clinical exposure is undertaken subsequently in the fifth year, which includes cavity preparation and composite resin restoration placement. However, even following their clinical experience, sixth‐year students sometimes give incorrect answers about the procedures to follow in these cases during their examinations. Although they have gained a basic understanding of these procedures during classroom teaching, it appears that they may have failed to retain that knowledge during their clinical training. One of the reasons for such rapid loosing of their knowledge may be associated with the way of their study method. Students concentrate on multiple‐choice questions (MCQs), because JNE is performed with MCQ, and it is getting more difficult to pass the JNE. MCQ exam is convenient for teachers, because we can evaluate the level of many students in a short period. But it is also necessary for us to find the level of each students in a small group learning. To overcome this problem, we developed a new test to evaluate students' knowledge and provide motivation for continuing study in Year 5.

The current structure of the curriculum requires fifth‐year students to participate in clinical training almost every day, with the result that there is only a 1‐hr slot available for supportive didactic teaching. Given the broad coverage of the field of conservative dentistry, it is recognized that there are limitations as to what can be addressed in 1‐hr segments, and the provision of MCQ tests may not improve motivation for study. Therefore, an “error‐detection examination” for students was developed for this cohort to investigate their perceptions of this new active‐learning method. This report outlines the error‐detection examination and presents the results of students' evaluation of the process.

## 2 METHODS

### 2.1 Participants and clinical training

Participants were fifth‐year students at Fukuoka Dental College. In total, 86 students performed clinical training under the guidance of instructors. The students need to experience all the clinical field in half year. They were divided into four groups of 21 or 22 students, and each group visit clinical department, such as Dentistry for the Disabled,

Pain Clinic, General Dentistry/Halitosis Clinic Center, Geriatric Dentistry/Visiting Dentistry, Operative Dentistry & Endodontics, Periodontics, Prosthodontics, Oral Implant, Orthodontics, Oral Surgery/Sports Dentistry, and Pediatric Dentistry.

At the clinic of General Dentistry, about 20 instructors supervised them performing dental treatment, including restorations. The students need to complete cases, which are indicated in the clinical training manual, and the instructors checked the cases according to the instructor's manual.

### 2.2 Error‐detection examination procedure

Each group performed clinical training for 15 days at the Department of General dentistry. On the last day, after the clinical training, each group of students gathered in the seminar room of our department, where we distributed error‐detection examination sheets. These sheets presented detailed written treatment procedures for typical cases, but some steps were purposefully written incorrectly. Students had to identify and amend these incorrect steps in the procedures. After correcting them, each student gave a presentation on their corrections to the group. This was followed by delivery of a summary of the ideal answers and a short lecture by a teacher.

### 2.3 Case reports used in the error‐detection examination

Cases on local anesthesia, composite resin filling, amalgam removal, and ceramic inlay restoration were prepared for the error detection examination. Examples of the cases are shown in Appendix A.

### 2.4 Students' evaluation

After the teacher's summary of correct answers and lecture, an anonymous questionnaire was distributed to the students to investigate their perceptions of the examination (Appendix B). Only completed questionnaires were analyzed. There were 85 valid responses, giving a valid response rate of 98.8%. All students provided consent for analysis and publication of their responses.

### 2.5 Ethics

This work was conducted in accordance with the Declaration of Helsinki. All participants understood the nature of this research study and provided written informed consent to participate. Permission for this study was obtained from the Ethics Committee for Clinical Research of Fukuoka Dental College and Fukuoka College of Health Sciences (approval No. 390).

## 3 RESULTS

### 3.1 Errors students could not detect

The percentage of students who could not identify the errors is shown in Table 1. We found that many students could not detect incorrect procedures relating to ceramic inlay treatment. More than 60% of them could not obtain a correct answer for the occlusal adjustment of ceramic inlay, and 52.9% of students could not identify the incorrect cementation processes for a temporary restoration after ceramic inlay cavity preparation.

**Table 1:** The number of students (and percentage) who could not identify the error

Question #	Procedures	Number of students with error (%)
1	Local anesthesia technique	18 (21.2)
2	Caries removal with steel bur	27 (31.8)
3	Cavity preparation for composite resin filling	33 (38.8)
4	Composite resin filling	6 (7.1)
5	Amalgam removal	13 (15.3)
6	Cavity preparation for ceramic inlay	28 (32.9)
7	Temporary filling of ceramic inlay cavity	45 (52.9)
8	Contact strength of class 2 ceramic inlay	9 (10.6)
9	Adjustment before ceramic inlay set	52 (61.2)
10	Bonding of ceramic inlay	33 (38.8)

More than 30% of students forgot the basic procedures of cavity preparation.

### 3.2 Results of the students' evaluation

**Figure 1:**
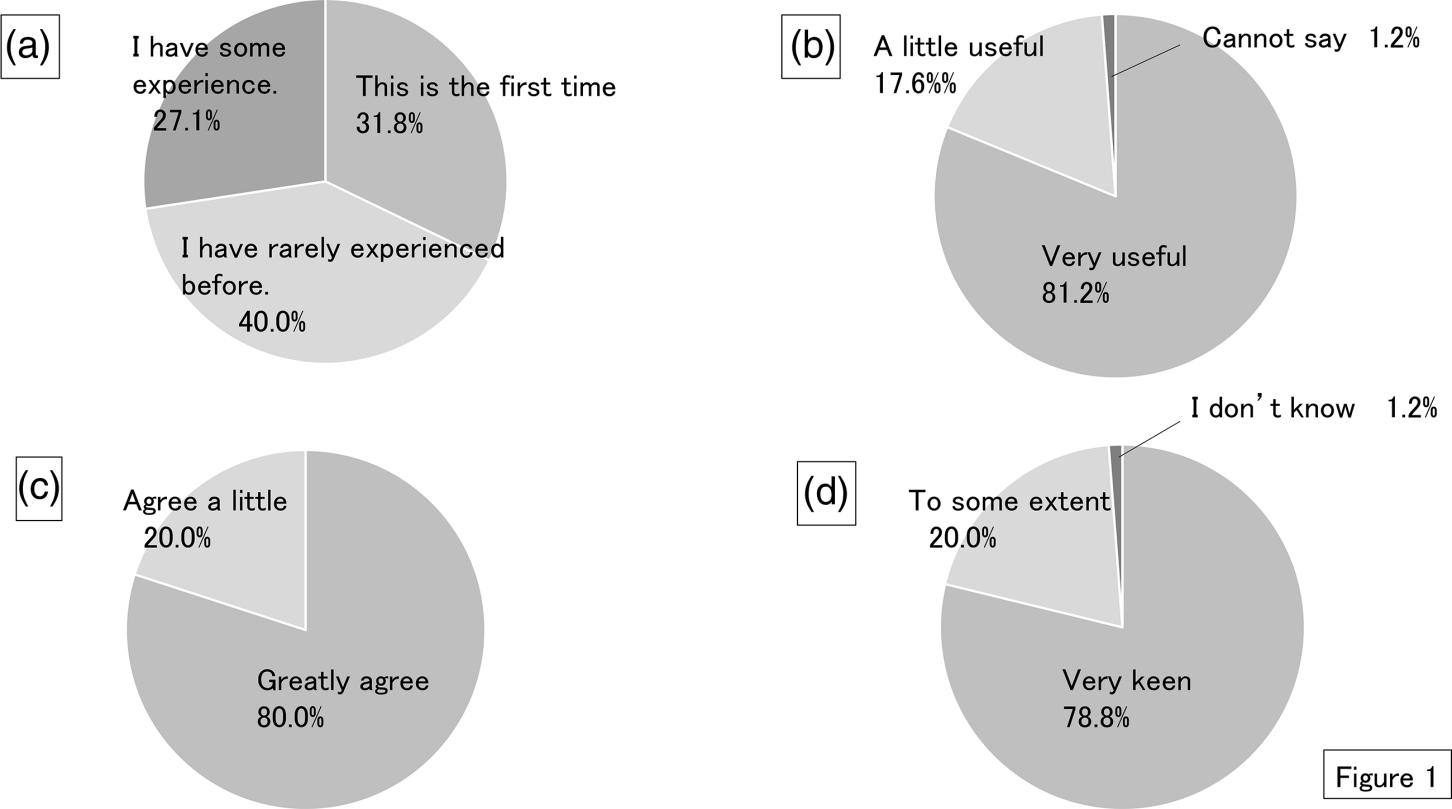
Pie chart illustrating the results of the questionnaire. (a) Have you previously experienced this kind of teaching? (b) Was the exam useful in confirming the level of your knowledge? (c) Were you motivated to revise conservative dentistry again? (d) Do you want to complete this form of teaching again?

The results of the evaluation questionnaire are shown in Figure 1. More than 70% of students indicated this type of error‐detection examination was new to them. In addition, about 81% said the test was useful to show them their level of knowledge. After the error‐detection examination, 80% of students also reported that they wanted to study conservative dentistry thoroughly again. In addition, nearly 80% of students hoped this type of test would continue.

### 3.3 Students' impressions and opinions

We invited students to provide free‐text comments about the error‐detection examination (Appendix C). Most comments were positive, and students appeared to have enjoyed the examination. Participating students also offered important suggestions, such as using a photograph‐based examination.

## 4 DISCUSSION

As it deals with preservation of tooth structure, involving preventive and reparative techniques, conservative dentistry is a crucially important field in clinical dentistry. Errors in restoration may lead to secondary caries resulting in pain and may contribute to a reduced lifetime of a repaired tooth (Brouwer, Askar, Paris, & Schwendicke, 2015). Therefore, to improve the care of patients, a deep understanding of conservative dentistry is important, and this is reinforced by the requirement for students to have complete knowledge of all treatment procedures to pass the JNE.

In some Japanese dental schools, dental students in the third and fourth years receive lectures on conservative dentistry and practice dental treatment with a simulator. In the fifth year, they attend clinical training and treat patients under the guidance of instructors. However, despite having had the clinical experience to reinforce their learning, in their final year, just before sitting the JNE, it is observed that some make mistakes in conservative dentistry knowledge tests. One reason for these mistakes may be a failure on their part to revisit their basic knowledge during their clinical training, due to their concentration on technical skills and communication with patients. During this period, they may not feel the need to refer to educational material to refresh their knowledge, as they had previously passed the relevant assessments. In addition, when they study, they concentrate on MCQ exam, because JNE is performed with MCQ exam. We developed a new strategy to overcome this problem, and reactivate prior knowledge, using an error‐detection examination.

The intention of this was to improve students' understanding of important points in the provision of clinical dental treatment. We developed scenarios that simulated clinical treatment and contained important points for dental treatment. Some procedures were purposefully written incorrectly, and students were required to find and correct these errors. Students found some errors easily, but some students could not identify important errors or missed key points that were important for dental procedures during clinical training. In addition, it became clear that knowledge necessary for the JNE was lacking, including some points that teachers had previously emphasized and which had been assessed previously as being sufficient.

When the fully correct answers were finally presented by a teacher, students became aware that they had forgotten important points or had incompletely remembered some procedures, and we considered that this was an important process to help consolidate deep learning by the student. There are many steps in restorative treatment, and students need to have a detailed understanding of these procedures. Our error‐detection examination appeared to have alerted students to points that they had forgotten or misunderstood.

In the evaluation questionnaire, participating students indicated that this type of examination was new to them and had helped to highlight their lack of knowledge in certain areas. They also said that this form of active learning had increased their motivation to study conservative dentistry. In this way, we could obtain the additional educational effect to the regular teaching with MCQ exam. Some students offered positive suggestions for future formats, such as using photograph‐based examinations. This pilot of an error‐detection examination did not include photographs of dental treatment, but we plan to use photograph‐based scenarios for the cohort group of students. It is hoped that the use of images will more accurately simulate clinical situations and help in preparation for the JNE. We are also thinking of comparing the result of the questionnaires of this time and the new type exam including photograph‐based scenarios. The statistical analysis of these results will make the educational effect clearer.

We found that the error‐detection examination was also beneficial for teachers, as it helped to identify common areas of students' misunderstanding and allowed use of this information to improve lectures. We were surprised to know that the students lack full knowledge of ceramic inlay treatment, although this may be because metal inlay treatment is more common experienced in our clinics. This has highlighted the need to for us to increase the opportunity to show cases of ceramic inlay to the students.

As in other health care areas, the practice of dentistry undergoes continuous development, and we need to monitor the progress of each student and identify areas of relative weakness in dental education (Patel, Fox, Grieveson, & Youngson, 2006). Furthermore, as reflective practitioners, academic dental educators play a major role in training future dentists and need to continuously improve their own teaching skills (Kérourédan et al., 2018). Previous studies have also indicated that questionnaire surveys to measure students' understanding were effective in improving clinical teaching (Youngson, Fox, Boyle, Blundell, & Baker, 2008).

Several different strategies are available for the delivery of teaching, including lectures, case discussions, questioning, feedback, reflection, and suggested reading (Foty, Gibbs, Lips, Menon, & Hafler, 2018). Although lectures are the most common teaching style and are good for primary explanations and clarifying concepts, this method is teacher‐oriented rather than learner‐oriented (Manning, Abrahamson, & Dennis, 1968). Case discussion is useful for problem‐solving and critical thinking but is less easy to deliver to a large cohort of students as it requires a large amount of teaching time. It is widely recognized that feedback is a very important part of the learning and teaching process as it brings about changes in behavior, which is essential for learning and may be the most tangible evidence that learning has been adopted deeply by the student. Questioning is effective in broadening ideas, but skills are required to understand the range of question types. MCQs are considered useful for efficiently judging knowledge (Haladyna, 1992), especially when followed by good feedback (Jang & Marshall, 2018).

Finally, descriptive tests are able to confirm the depth of knowledge. However, error‐detection examinations cultivate students' insight, and one Japanese university has recently introduced this form of test in Information and Communication Technology‐based education (personal communication), and we feel that this is transferrable to dentistry.

We had limited time for teaching with this group of students, and we applied the error‐detection examination with brief feedback. As feedback is important for education (Shahzad, Humza Bin Saeed, & Paiker, 2017), we plan to allocate more time for feedback next year. To further improve the educational effect, we are considering reflection as another teaching strategy. Reflection can examine the aspect of experience, allows expression, and determines meaning (Koole et al., 2013). The present test was performed in small groups, which allowed us to identify the level of each student. Team‐based learning (TBL) is another method performed with small groups. We are considering applying this test to TBL, because TBL is known to have a different educational effect than conventional lectures (Nishigawa et al., 2017). It is also important to improve teaching skills (Kohls‐Gatzoulis, Regehr, & Hutchison, 2004), and we aim to further improve the error‐detection examination.

## 5 CONCLUSION

Although conservative dentistry is a basic field of dental treatment, mistakes in treatment can lead to early treatment failure or reduce the lifetime of a restored tooth. Therefore, students need to have a deep understanding of procedures. Error‐detection examinations may help students identify knowledge gaps and provide useful feedback to teachers to identify areas that they should stress in earlier years.

## CONFLICT OF INTEREST

The authors have no conflicts of interest relating to this study to declare other than the grants reported above.

## APPENDIX A

### A.1. A CASE OF CARIES REMOVAL AND COMPOSITE RESIN FILLING

Pt. A visited our hospital to have the cavity in the upper front tooth treated. Dr. B found a caries in the cervical area and decided to fill a composite resin there. He first injected anesthesia in ①the gingival papilla, then added anesthesia to the gingivolabial fold.

Softening dentin was stained with caries detector, and the Dr. B removed the carious lesion with ②a high‐speed diamond point.

The cavity was prepared as ③a box form, and composite resin paste was filled ④all at once.

### A.2. A CASE OF AMALGAM REMOVAL AND CERAMIC INLAY SET

Pt. C visited our hospital with a complaint of an esthetic failure in the lower molar filling. Dr. D decided to remove the old amalgam filling and to replace with ceramic inlay. The amalgam filling was removed ⑤without water spray, and a ⑥straight bevel was added to the ceramic inlay cavity. After taking impression, the cavity was temporarily filled with ⑦zinc‐oxide eugenol cement.

After 1 week, Pt. C came. The contact pressure of the ceramic inlay was adjusted, and Dr. D confirmed that the ⑧110 micrometer‐contact gauge could be inserted. Occlusal contact was ⑨extra‐orally adjusted. The ceramic inlay was finally set with a ⑩glass ionomer cement.

## APPENDIX B Content of the questionnaire survey

Please answer the following questions. Check one answer to each question.

Q1. How often have you experienced an error‐detection examination in your studies ?

- () This was the first time for me.
- () I have rarely experienced this form of teaching.
- () I have sometimes experienced this form of teaching.
- () I have often experienced this form of teaching.

Q2. Was the error‐detection exam useful in confirming the level of your knowledge?

- () It was very useful.
- () It was a little useful.
- () I cannot say whether it was useful or not.
- () It was not particularly useful.
- () It was not useful at all.

Q3. Did you think your experience of this examination has motivated you to revise your study of conservative dentistry?

- () I was motivated to revise my study thoroughly.
- () I was motivated to study again to some extent.
- () I cannot say whether this has affected my intention to revise the subject.
- () I was less motivated to study again.
- () I was discouraged from further study.

Q4. Do you want to experience this form of teaching again?

- () I am very keen to.
- () I want to, to some extent.
- () I don't know whether I want to or not.
- () I am not very keen.
- () I do not want to.

## APPENDIX C Students' free‐text comments

- This is the first type of test, and I was interested in the test.
- I found that I had incompletely remembered steps of treatment.
- I think I can apply in clinic.
- I want to try this test every week.
- Please make tests in other field of dentistry.
- It was a good opportunity to review my on‐the‐job training.
- It is also important to solve questions other than multiple choice question.
- I was not fond of conservative dentistry before, but I enjoyed the test.
- I wanted to try more test.
- Please include visual materials next time.
